# Genetics of Autosomal Recessive Spastic Ataxia of Charlevoix-Saguenay (ARSACS) and Role of Sacsin in Neurodegeneration

**DOI:** 10.3390/ijms23010552

**Published:** 2022-01-04

**Authors:** Jaya Bagaria, Eva Bagyinszky, Seong Soo A. An

**Affiliations:** 1 Department of Bionano Technology, Gachon University, Seongnam 13120, Korea; jbagaria1206@gmail.com; 2 Department of Industrial and Environmental Engineering, Graduate School of Environment, Gachon University, Seongnam 13120, Korea

**Keywords:** ARSACS, sacsin, neurodegeneration, ataxia, mutation

## Abstract

Autosomal recessive spastic ataxia of Charlevoix-Saguenay (ARSACS) is an early-onset neurodegenerative disease that was originally discovered in the population from the Charlevoix-Saguenay-Lac-Saint-Jean (CSLSJ) region in Quebec. Although the disease progression of ARSACS may start in early childhood, cases with later onset have also been observed. Spasticity and ataxia could be common phenotypes, and retinal optic nerve hypermyelination is detected in the majority of patients. Other symptoms, such as pes cavus, ataxia and limb deformities, are also frequently observed in affected individuals. More than 200 mutations have been discovered in the *SACS* gene around the world. Besides French Canadians, *SACS* genetics have been extensively studied in Tunisia or Japan. Recently, emerging studies discovered *SACS* mutations in several other countries. *SACS* mutations could be associated with pathogenicity either in the homozygous or compound heterozygous stages. Sacsin has been confirmed to be involved in chaperon activities, controlling the microtubule balance or cell migration. Additionally, sacsin may also play a crucial role in regulating the mitochondrial functions. Through these mechanisms, it may share common mechanisms with other neurodegenerative diseases. Further studies are needed to define the exact functions of sacsin. This review introduces the genetic mutations discovered in the *SACS* gene and discusses its pathomechanisms and its possible involvement in other neurodegenerative diseases.

## 1. Introduction: Sacsin (*SACS*) Gene and ARSACS

The sacsin gene (*SACS*) is located on chromosome 13 (13q12.12: chr13:23,288,689-23,433,763, GRCh38/hg38), with 145,075 bases, and is oriented in the minus strand of DNA (https://www.genecards.org/cgi-bin/carddisp.pl?gene=SACS accessed on 24 December 2021). *SACS* comprises 10 exons, with nine coding exons, and the 10th exon contains 11,487 base pairs, notable as the longest exon among vertebrates [1,2]. The *SACS* gene encodes a large 520-kDa multidomain protein of 4579 amino acids, called sacsin. It contains several different domains, including the ubiquitin-like (UBL) domain in the N-terminal region, three sacsin internal repeat (SIRPT or SRR) domains, the helical XPC-binding domain, a sacsin J-domain and the higher eukaryotes and prokaryotes nucleotide-binding (HEPN) domain in the C-terminal region [3]. Sacsin is expressed in several different tissues, with higher expression in the central nervous system or skin and lower expression in the pancreas and skeletal muscle. In the brain, sacsin expression is the highest in the motor system, including the cerebellum, granular system and in Purkinje cells [2].

*SACS* is associated with early-onset cerebellar ataxia due to mutation, called the spastic ataxia of Charlevoix-Saguenay (ARSACS), in an autosomal recessive pattern; it was first discovered in a population by the linkage disequilibrium studies conducted in the Charlevoix-Saguenay-Lac-Saint-Jean (CSLSJ) region in Quebec [4,5]. Based on several relatives with ataxia syndrome, the founder effect was present in the French Canadian population. Since this family immigrated to French Canada in the 17th century from the Perche region, it is possible that the disease was present in France, although similar cases may remain unrecognized. Other cases of *SACS* ataxia in non-Quebec populations, such as in Italy, Japan, Spain, Tunisia or Turkey, were discovered [6,7,8].

ARSACS is associated with ataxia, dysarthria, nystagmus, spasticity, distal muscle wasting and deformities in fingers or feet [6,7]. Affected patients show slow progression of spastic ataxia, which may affect all four limbs. Patients also experience a loss of muscle tissue (amyotrophy) and language impairment (their speech became slurred). Ocular movements may also be impaired [6,7]. Disease may occur during childhood (lower limb ataxia), but their intelligence may not be impaired. Cerebellar signs and pes cavus appear until patients reach their 20 s. Other characteristics may also be possible, such as retinal nerve fiber hypermyelination [8,9,10]. The first characteristics of the disease may be gait initiation, which can be noticed when children start to walk (around 12–18 months of age). Childhood ataxia and spasticity may be prominent as well. In early adulthood (20 s or 30 s), disease progression may be accelerated, and patients may lose the ability to walk by the age of 50 [9]. Bladder and bowel dysfunctions could appear in patients in their 50 s [10]. In addition, patients may show biochemical dysfunctions, such as impaired pyruvate oxidation, hyperbilirubinemia and low serum beta- or HDL lipoproteins [11]. In patients with ARSACS, non-phosphorylated neurofilaments (NFs) may occur in different neurons, including Purkinje cells or motor neurons in the cortex. The cultured motor neurons of *SACS* knockout mouse embryos presented abnormal neurofilament rearrangements [12,13]. Abnormal mitochondrial functions were also detected, with lower mitochondrial motility and elongation. Since NFs could impact cytoskeletal organization, the alteration of NFs in patients with ARSACS may present mitochondrial impairments, resulting in elevated cellular vulnerability [12,13]. In this review, mutations in the *SACS* gene in patients with ARSACS and the potential involvement of sacsin in other forms of neurodegeneration are discussed.

## 2. Sacsin Functions and Cell/Animal Models of ARSACS

One of the limitations in understanding the role of *SACS* in ARSACS was the unavailability of brain tissues from the affected patients. Ideal cell/animal models should provide insights and reduce the time and costs of analysis, which could be used in screening drug candidates in preclinical studies [14]. To date, several cell and mouse models (hiPSC, SH-SY5Y, knockout mouse models) have been developed with high priority in ARSACS to reflect the accurate human conditions for the discovery of the disease mechanisms. However, the differences in genome, anatomy and metabolisms between humans and mice could represent limitations of mismatch in the ideal replication of the disease [2,13,15]. *SACS* knockout mice, investigated by Larivière (2015), revealed typical ARSACS symptoms, loss of Purkinje cells in the cerebellum and abnormal aggregation on non-phosphorylated neurofilaments in the somatodendritic brain region with altered mitochondrial dynamics [12]. An additional study on *SACS* –/– mouse Purkinje cells by Ady et al. showed that *SACS* knockout cells could reduce the firing rate, lower the excitatory synaptic rate and lead to the loss of Purkinje cells in the anterior lobes, but not in the posterior ones [15]. Loss of Purkinje cells was prominent in the deep cellular nuclei, and the disturbances appeared in the cerebellar circuit [15]. Human-derived cell models may mimic more accurately the human-related pathways and could be more effective in studying disease-related mechanisms and therapies [16]. Human-induced pluripotent stem cells (hiPSC) were suggested to open new avenues in disease modeling and also drug development. They may mimic more correctly the disease mechanisms, even from the earliest cellular dysfunctions. However, the challenge with hiPSC could be that they may not reflect the complexity of different brain areas (cerebellum in ARSACS). An additional issue could be the need for protocols to be developed for an appropriate ARSACS model, such as creating more humanized cell lines, providing nutrients to cells. With hiPSC cells, it may be difficult to model aging-related diseases [16]. SH-SY5Y cells with sacsin knockout may also be a promising approach to monitor the alterations of gene/protein expression or cellular changes [17,18]. Since SH-SY5Y are widely used cell lines in neuroscience and are low-cost human-derived cell lines, these can be used to differentiate various neuronal phenotypes [18]. However, the issues with SH-SY5Y cells are that no standardized protocols are available on them to maintain the culture and to grow the neuron of interest [14]. The studies by Duncan et al. (2017), Crisuolo et al. (2015) and Girard et al. (2012) on human dermal fibroblasts (HDFs) from ARSACS patients revealed reduced sacsin expression. Lower sacsin expression was associated with impaired mitochondrial functions, IF (vimentin) aggregation, abnormal chaperon activity and increased autophagy [19,20,21]. HDFs could be a convenient disease model without genetic engineering to study the disease mechanisms of several human diseases. HDFs have been used to study several neuropsychiatric disorders [22,23]. However, isolation and culture techniques in HDFs are time-consuming and require materials and extensive studies to improve the optimization of the protocols [24]. Taken together, promising studies have been performed to analyze sacsin functions and ARSACS disease mechanisms in cell or mouse models, but further research is needed on the ideal disease model organism.

## 3. Sacsin Protein Domains

**Figure 1:**
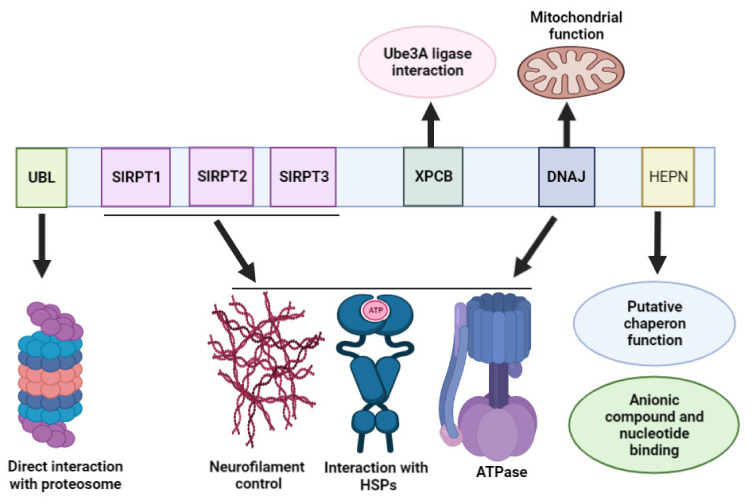
Schematic showing the domain structure of the of sacsin protein and the potential functions of different domains.

Sacsin has five domains: ubiquitin-like domain (Ubl), three large sacsin internal repeats (SIRPT1, SIRPT2, SIRPT3), xeroderma pigmentosum C-binding domain (XPCB), J-domain (DNAJ) and higher eukaryotes and prokaryotes nucleotide-binding (HEPN) domain. These domains could have different functions and interacting partners (Figure 1). However, they also could also cooperate in different pathways [25].

The N-terminal region of sacsin could interact with proteasomes. Additionally, it may play a role in regulating protein folding by interacting with heat shock proteins. The Ubl domain could interact directly with the proteasome system through the 19S cap and 26S proteasomes and be involved in the degradation pathway [2,26]. The SIRPT domains contain a homologous region, the Hsp90 chaperone [5,13,27,28], which was suggested to be involved in ATPase activities. ATP hydrolysis is important for proper sacsin function. Dysfunctions of the SIRPT domain were associated with reduced or lost ATP hydrolysis. Along with the J-domain, SIRPT domains could stimulate the ATPase activity of Hsp70 [28,29]. The XPC-binding domain (XPCB) could bind the Ube3A ubiquitin ligase. It may be possible that dysfunctions in Ube3A–sacsin interaction could impact the onset of Angelman syndrome-related ataxia [30].

The J-domains can enhance the protein–protein interactions and regulate the activity of heat shock proteins, including Hsp70. A J-domain contains Hsp40 homologous sequences and may impact homeostasis. An interaction between sacsin, Hsp70 and the ubiquitin proteosome system may be involved in defensive mechanisms against abnormal protein aggregation [2]. Sacsin was suggested to play a role in controlling the homeostasis between intermediate filaments (IFs) and neurofilaments (NFs) [3]. If sacsin is missing, nerve cells could contain abnormal bundles of NFs. Patient-derived HDF cells showed abnormal IF (vimentin) distribution and broken microtubule organization. In ARSACS cells, the misfolded IFs aggregated and formed a cage-like structure around the microtubule organization center. These aggregates may result in abnormal clearance and autophagy [20]. Adding the SIPRT and J-domain into motor neurons from sacsin knockout mice resulted in a reduced amount of NF bundles. Both domains could prevent the assembly of NFs [3]. Treatment with SacsJ-myc-TAT in SACS+/+ motor neurons resulted in the induction of IF and NF disassembly. In Sacs–/– motor neurons, the NF bundles were resolved, and the NF network was restored [21]. These data suggest that the J-domain could impact the regulation of IF-NF assembly and disassembly directly [3,20,31].

The function of the HEPN domain was initially unclear [27], but it was found in different eukaryotes and prokaryotes. In bacteria, the HEPN region could impact antibiotic resistance (for example, kanamycin). In eukaryotes, the HEPN domain may have nucleotide-binding activity, but it could also bind anionic compounds in neurons [22,23,32,33]. HEPN domains could dimerize and form a high-affinity site for GTP binding, and potentially impact the chaperon activity of the sacsin protein. The HEPN domain may be involved in the elevation of the ATP/GTP concentration, and in the sacsin–Hsp70 interaction. The HEPN domain may also co-operate with the J-domain, and they contribute to nucleotide binding. Mutations in the HEPN region were suggested to disrupt the nucleotide-binding activity and result in abnormal folding/oligomerization of sacsin [3,27,32,33].

**Figure 2:**
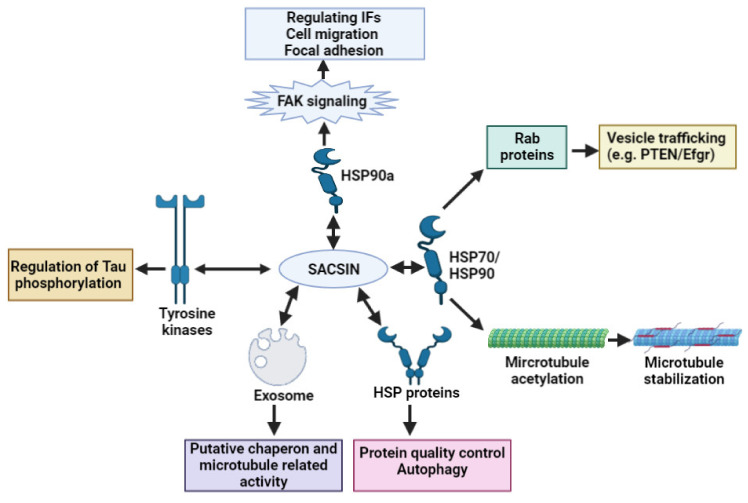
Possible impact of sacsin in different cellular processes, including chaperon functions, microtubule organization and vesicle trafficking.

A recent study by Romano et al. [17] revealed that, besides controlling the filament architecture, sacsin could play a crucial role in cell adhesion, microtubule organization and trafficking proteins. The mentioned authors provided an extensive study on the possible functions of sacsin. Knockout of the *SACS* gene in SH-SY5Y cell lines revealed several disease-associated changes. Microtubule organization and dynamics were altered in KO cells, microtubule polymerization was enhanced, and the movement of tubules became abnormal. Sacsin may also regulate Tau phosphorylation by interacting with tyrosine kinase enzymes. Several kinesin proteins were hyperphosphorylated, resulting in disturbances in mitochondrial movements. Tau and STMN1 pS16 were also hyperphosphorylated. These findings revealed that sacsin could possibly interact with tyrosine kinases. Additionally, sacsin interactions with HSP proteins could be critical in microtubule organization and stabilization. Non-functional sacsin also resulted in disrupted focal adhesion and dynamics, which could result in disturbances in axonal growth, synaptic formation and balance in the brain. Focal adhesion kinases were suppressed in *SACS* KO cells, and modulation of FAK-PTEN pathways may be beneficial in the case of cellular deficits. Disturbances were found in the adhesion mechanisms, since the membrane-bound adhesion molecules and cell adhesion molecules were both mis-localized. These findings suggest that, besides abnormal trafficking and cellular interactions, localizations could contribute to the reduced interaction between heat shock proteins and sacsin, leading to ARSACS pathology. Additionally, sacsin may interact with exosomes, which could also impact several chaperons or microtubule-related mechanisms. Figure 2 demonstrates that sacsin may serve as a critical regulator of different cellular processes, including chaperon activity, the transport of vesicles and microtubule and filament organization [17].

**Figure 3:**
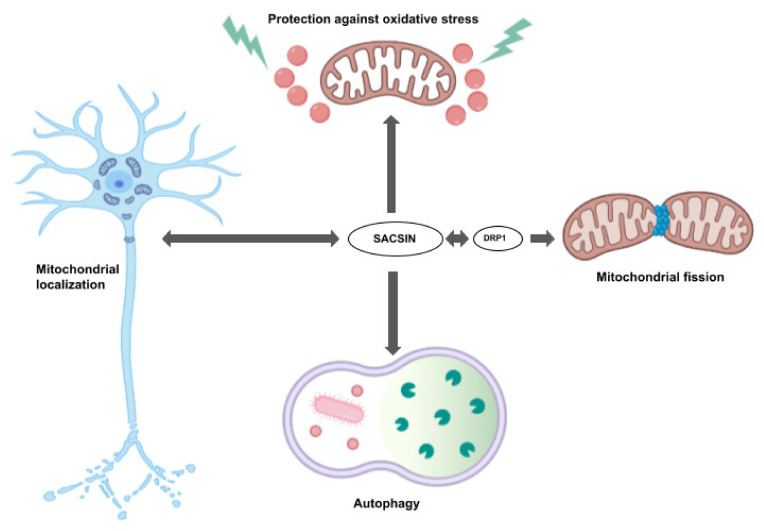
Sacsin involvement in mitochondrial functions.

Sacsin is also localized in mitochondria and was suggested to play a key role in different mitochondrial functions (Figure 3). It was suggested to be involved in mitochondrial network regulation and connection and morphology. Sacsin was verified to bind the dynamin-related protein 1 (Drp1) [21]. Lower sacsin levels resulted in failure to recruit appropriate amounts of Drp1, leading to abnormalities in mitochondrial quality control [34]. Cellular and animal model studies were investigated to define the pathways related to sacsin dysfunctions. Girard et al. (2012) found that fibroblasts from ARSACS patients presented abnormal mitochondrial functions [31]. Later, Pilliod et al. suggested that monitoring mitochondrial abnormalities could be a possible diagnostic biomarker in ARSACS patients [35,36]. Sacsin also plays an important role in controlling the localization of mitochondria in neurons, and in appropriate dendrite development and morphology. Knockdown of *SACS* could result in several mitochondrial dysfunctions, such as abnormal hyperfused/balloon-like/bulbed mitochondria, or an abnormally interconnected network through lower fission. Additional dysfunctions could be disturbances of mitochondrial transport into neurons. Mitochondria accumulated in soma and proximal dendrites instead of the inner neural cell bodies and along the full length of dendrites. In nerve cells with non-functional sacsin, the number of dendrites was reduced. In addition, the remaining dendrites became thicker compared to the normal ones [21]. HGF studies by Criscuolo et al. revealed that sacsin dysfunctions could reduce the mitochondrial respiratory rate and ATP synthesis, resulting in oxidative stress [19]. Morani et al. used Crispr/Cas9 technology to knockout sacsin in SH-SY5Y cell lines, which showed that cells with *SACS* knockout obtained reduced oxygen consumption and higher DNA damage by reactive oxygen species (ROS). The gene expression profile was also changed: differently expressed genes were observed in several pathways, such as autophagy, mitochondrial dynamics and apoptosis [36]. There is a possibility that sacsin may also influence autophagy. Morani et al. (2019) analyzed differentially expressed genes (DEGs) in sacsin knockout SH-SY5Y neuroblastoma cells, and, besides oxidative phosphorylation and mitochondrial dynamics, the autophagy- and cell-death-related genes were included among the most significant DEGs. Cells without functional sacsin showed a reduced degree of autophagosome aggregation and its fusion for lysosomes. These findings also reveal that ARSACS dysfunctions could result in reduced clearance of damaged cellular organelles [36].

## 4. *SACS* Genetics and Mutations

**Figure 4:**
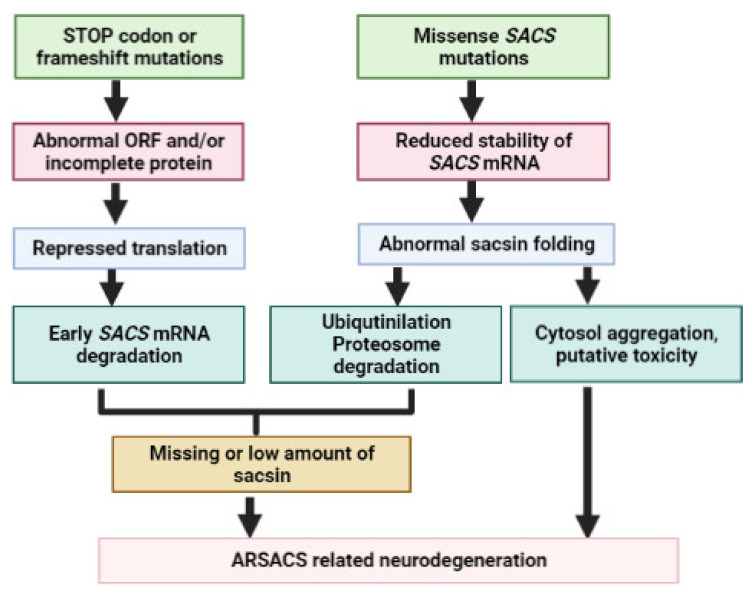
Potential effects of mutations in *SACS* gene.

More than 200 pathogenic mutations have been reported in the *SACS* gene all around the world (Table 1, Table 2, Table 3 and Table 4). The majority of mutations were found in exon 10, which was verified as the longest exon among vertebrates [37]. The majority of mutations may result in non-functional *SACS* or reduced sacsin expression. Disease-associated variants can be either homozygous or compound heterozygous. The phenotypes of mutations may be diverse; besides the classical phenotypes (ataxia, spasticity), additional atypical symptoms may also be present (mental retardation, memory dysfunctions). Mutations can be either missense mutations, STOP codon mutations or frameshift variants (Figure 4). The missense mutations could result in lower stability and abnormal conformation of sacsin. Normally, the misfolded sacsin goes through co-translational ubiquitination and degradation by the proteosome system. If degradation does not happen, mutant sacsin could potentially aggregate in cytosol. Based on other neurodegenerative diseases, the potential aggregation of sacsin may result in putative additional gain-of-function toxicity. The frameshifts or nonsense variants may repress the translation, resulting in the degradation of the *SACS* transcript. All of the above could be associated with missing sacsin expression or insufficient amounts of sacsin protein [38].

The first *SACS* mutations were reported in the CSLSJ family. In this population, more than 300 patients were observed who could have been the descendants of a single founder [5,37]. Several mutations were found in the affected population in the Quebec region. Initially, two pathogenic mutations were identified, c.6594delT (mutation location on transcript) or p.I2949Ffs*4 (mutation location on protein, 94% of mutant alleles) and c.5254C>T (p.Q1752X, 3%). Both mutations could be related to the truncation of the sacsin protein [5,37,38]. Later, additional variants were observed in the Quebec population, including five missense variants, five indels, one nonsense and one large genome deletion. The mutation c.8844delT (p.I2949FfsX2952) seemed to be relatively common among ARSACS patients. However, additional variants also appeared in several ARSACS cases, such as c.4744G>A (p.D1582N), c.814C>T (p.R272C), c.237insAfsX (p.S80IfsX98) and c.5836T>C (p.W1946R). This study also suggested that large deletions in French Canadians (Table 1) may not be either common or a lethal variant. The diverse phenotypes of the disease may appear due to the partial loss of function in the sacsin protein [39,40,41].

**Table 1:** *SACS* mutations detected in French Canadian and Tunisian patients. “hm” means mutation carried the homozygous form, “c het” means mutation had compound heterozygous allele. “rs” means respectively.

Mutation	Exon	Domain	AOO	Clinical Symptoms	Neurological Changes	Population (Ethnicity)	Refs.
c.6594delT (I2949Ffs*4,) or c.5254C > T (p.Q1752X), hm or c-het	10	SIRPT1 + SIRPT3 rs	12–18 mths	ataxia neurotrophy learning disability mental dysfunctions foot deformities	cerebellar loss of Purkinje cells cortical degeneration	Canada (French Canadian)	[5,38]
several variants, c.8844delT (p.I2949FfsX2952): most common, hm or c-het	8 or 10	SIRPT1, 2 or 3, XPBC or DNAJ	NA	NA	NA		[42]
c.9284dupC 9p.Ala3096Cysfs*2), hm	10	SIRPT3	early child- hood	clumsy gait uncoordinated hand movement mild non-progressive dysarthria intermittent dysphagia pes cavus	atrophy of cerebellar vermis		[41]
c.10046G > C (p.A3324P), hm	10	Between SIRPT3 and XPBC	2–4 yrs	ataxia gait dysfunctions spasticity	cerebellar syndrome cerebellar dysarthria no myelinated nerve fibers	Tunisian (Tunisian)	[43]
c.1411delT, hm	8	SIRPT1	5 yrs	ataxia gait dysfunctions			
c.1155insA, hm	8	SIRPT1	1–9 yrs	ataxia gait dysfunctions pyramidal syndrome			
c.3662T > C (p.W1196R), hm	10	SIRPT1	10–14 yrs	ataxia gait dysfunctions			
c.12846_12850delAGAG, hm	10	Between XPBC and DNAJ	1–3 yrs	spastic gait ataxia dysarthria unsteadiness	horizontal nystagmus neuropathy no retinal hypermyelination		[44]
c.2439-2440delAT(V815Gfs*4), hm	10	SIRPT1	13–19 yrs	ataxia nystagmus	no loss of large myelinated fibers		[45]

Besides French Canadians, several *SACS* mutations and ARSACS cases have been detected around the world. In Tunisian families, patients with ARSACS were late-onset cases with similar clinical symptoms in comparison to the French Canadians [43,44]. In 2003, 18 ARSACS patients from four families in Tunisia were analyzed and displayed two STOP codon and two missense mutations: c.10046G>C (A3324P), c.1411delT (frameshift, resulting in premature STOP codon of residue 456), c.1155insA (frameshift, resulting in premature STOP codon of residue 360) and c.3662T>C (W1196R) (Table 2). The STOP codon variants were predicted to be involved in the loss of DNAJ domain of the sacsin protein and reduced or loss of chaperon activity. The missense mutations could result in abnormal folding of the protein secondary structure. Although these families were not related to each other, inbreeding was found to be high in Tunisia, suggesting the possibility of a founder effect [43]. Furthermore, in 2009, an additional mutation was found in a Tunisian male patient with a familial case of ARSACS who developed dysarthria and gait dysfunction between one and two years of age, followed by language impairment and ataxia. A four-base deletion was found in proband c.112846_12850delAGAG, resulting in a STOP codon at residue 4305. Parents of patients were first cousins and carried the heterozygous indel. The mutation was associated with a loss of 274 amino acids and missing C-terminal region, including the DNAJ domain [44].

In Europe, several ARSACS cases were discovered in different countries, including Italy, Spain, Turkey and Belgium (Table 2) [46]. In Italy, ARSACS has been quite extensively studied. The first cases of *SACS* mutations were discovered by Grieco et al. (2004), and this study reported three novel variants in patients. The first patient carried a 5-base deletion (del4999cagaa5003) at residue 4999, which could result in the absence of the most protein sequence, including the DNAJ and HSP90 domains. The second patient carried two possibly pathogenic variants: 1858C>T (Q620X) and an insertion (4585insA: V1528fsX1540) [47]. Another Italian mutation was discovered in SACS, 1859insC, resulting in a premature STOP codon at residue 599, where most of the protein was lost [48]. Bi-allelic cases also appeared in Italian patients, such as c.563G>A (p.G188Q) + c.7394C>T (p.S2465L) double mutations and c.962G>A (p.R321Q) + c.8330G>A (p.R2777K) double mutation in two different Italian patients, and both were predicted to be damaging in silico. Both patients presented elevated levels of mitochondrial fragmentation. The patient with c.563G>A (p.G188Q) + c.7394C>T (p.S2465L) mutations also showed reduced sacsin levels in fibroblasts [49]. In 2013, Prodi et al. found several homozygous or compound heterozygous *SACS* mutations, and one of them was a large in-frame deletion. The majority of patients developed the first disease phenotypes at early childhood, but one of them presented late onset (32 years). These patients had early gait instability, Babinski sign and pes cavus, but spasticity and elevated muscle tone were not among the early symptoms. Imaging showed cerebellar superior vermis atrophy, pontine changes and thinning corpus callosum in all patients [50]. Additional homozygous variants also appeared in Italian patients, such as c.4198T>A + c.5719C>T [51], c. 1859insC [48], c.13132C>T [52], c.5629 >T [13], c.600_604+1delAACAGG [53], c.6680T>C [13] or c.10743C>T [54], c.11471A>G [54] and a large deletion at the *SACS* region. The majority of these cases were related to young-onset ataxia, and hypermyelinated retina was common among them. The patient with the homozygous c.600_604+1delAACAGG variant with the large 1.5 MB large deletion developed an ataxic phenotype at an atypically late age of 42 years [53]. Another compound heterozygous case of ARSACS mutations was found by Pensabene et al. (2020) in two siblings who developed ataxic symptoms before the age of 20, and it worsened in their 30 s [51]. Several *SACS* mutations were reported in Turkish cases. The first mutations were observed in 2004: this study reported two missense (W1196R and N3799D) mutations and two indels (L3193-fsX3199 and T2683-fsX2708)[55,56]. Later, additional mutations were also discovered: Oguz et al. analyzed nine unrelated families and revealed eight novel mutations in the *SACS* gene (5019A>G/F4011S, 14370G>T/S894X, 12841T>A/K1404X, 5031 G>A/ S4007F, 12660A>G/ I1464T, 8346–8347insT, 5677G>A/ R3801, 5566delC), and one variant (6945A>G/V3369A) [57]. Kurt et al. reported a homozygous G2772A mutation in a Turkish female patient with ataxia and spondyloepiphyseal dysplasia, but it was heterozygous in unaffected family members. The mutation co-existed with two homozygous mutations in the *ACAN* gene, which may have been responsible for skeletal deformations [58]. An additional mutation, c.2182C>T, appeared in a Turkish child with low progressive difficulty in walking and slurred speech. This child was negative for mutations responsible for any kind of spinocerebellar ataxia [59]. Recently, an indel (p.P4154QfsX20) was observed in a family with progressive spastic ataxia and dysarthria, with uncommon symptoms such as intellectual disability, hearing dysfunctions and epileptic seizures [60].

Besides Italy and Turkey, additional patients have been examined in other European countries. In the Dutch population, *SACS* mutations seemed to be quite prominent in patients with early-onset cerebellar ataxia. Vermeer et al. (2008) analyzed 43 families with ataxia. Patients developed a disease phenotype under 25 years of age. Among them, 16 patients showed novel mutations in the *SACS* gene in either the homozygous or compound heterozygous stage. Among these variants, there were eight STOP codon mutations, three were missense, two splice site variants and three deletions (in-frame or frameshift). Symptoms of mutations were very similar in each family: all of them developed early-onset ataxia (before 13 years of age) with lower limp spasticity, neuropathy in semi-motor axons and atrophy in the cerebellum [61]. The first Belgian case of *SACS* mutation was a missense variant, c.3491T>A (p.M1164K), detected in a family with similar symptoms to Quebec patients, but additional phenotypes also appeared, such as no retinal nerve hypermyelination or teenage onset of disease [62]. Baets et al. (2010) examined several ARSACS patients; the majority of them were Belgian descendants. In 11 families (17 patients), 18 mutations were observed, including an intragenic *SACS* exon 3-5 deletion. In 12 individuals, classic ARSACS phenotypes were found. Several patients had later onset of disease, since they developed gait difficulties or distal weakness after 20 years of age, which may not be associated with classical ARSACS [63]. The first mutation in Spain, reported by Criscuolo in 2005, involved c.7848C>T(p.R2556C), which presented a similar classical phenotype to the Quebec population. The mutation spared the DNAJ domain, co-segregating in the family, and was missing in controls. In addition, this variant was reported to affect a conservative residue (R2556), which also confirms its role in the disease [64]. Another Spanish female patient with a compound heterozygous p.R276C and p.P1302S was reported in 2015. She developed gait instability and slurred speech in her teenage years [65]. A Greek family carried a deletion, p.T3232KfsX24 (c.9695delC), where the siblings were homozygous for this variant, but their unaffected parents were both heterozygous. Affected siblings developed gait disturbances and ataxia in their first decade of life [66]. The first Polish case of ARSACS was described in 2017, in a patient who carried two novel mutations in the compound heterozygote stage: p.S3268_I3269fs/c.9804_9805insC and p.D4192N/c.12574 G>A. The mutation was missing in the healthy population, while unaffected siblings and the mother carried only one of these variants. Besides the classical ARSACS symptoms, affected patients presented cognitive or behavioral dysfunctions too [67]. A Russian case of the disease was discovered with atypically late onset of ARSACS and c.72276C>T (p.R2426X) mutation. The patient developed typical ataxia symptoms at the age of 32 years [68]. A Norwegian case of a compound heterozygous mutations c.13352T>C, p.L4451P; c.6890T>G, p.L2297W were found in a family with a typical form of spastic ataxia and other dysfunctions, such as cognitive decline and epilepsy [69]. In Germany, two studies were performed on ARSACS patients [4,70]. Synofzik et al. (2013) sequenced 22 patients with early-onset ataxia, and 17 novel homozygous or compound heterozygous variants were reported. All variants were missing in unaffected individuals and located in conserved domains. While some patients presented classical cases of ARSACS, some atypical symptoms were also apparent, such as pure neuropathy (which characterizes the Charcot–Marie–Tooth disease/CMT), missing neuropathy, absence of spasticity or cerebellar ataxia. Interestingly, besides the cerebellum and pons, MRI revealed abnormalities in other brain areas, such as atrophy of the cerebral parietal region or thinning of the corpus callosum [4]. Vill et al. (2018) identified seven *SACS* variants in nine patients who were diagnosed with non-syndromic hereditary motor and sensory neuropathy (HMSN). These mutations were associated with atypical phenotypes, such as sensory ataxia only, absence of spasticity and pyramidal signs. MRI also did not present any typical ARSACS characteristics, since it was either normal or presented isolated atrophy of the superior cerebellar vermis [70]. In the UK, a case was detected with unexplained multisystem neurological disorder in two siblings, who presented two heterozygous *SACS* variants (c.2076delG, p.Thr692Thr fs*713 and c.3965_3966delAC, p.Gly1322Val fs*1343). Patients had pyramidal signs, later onset (19–26 years) and no retinal nerve thickening or hypermyelination [71]. An additional study in the UK analyzed 191 patients with different types of genetic ataxia and 101 controls. Among them, 17 patients had ARSACS and carried 20 variants. Among these, 11 were unknown mutations. The majority of variants were compound heterozygous, but one deletion was found to be homozygous. The age of onset was variable (1–46 years). All of them had ataxia and nystagmus. The majority of them presented spasticity (expect one). Additional possible symptoms were sensory loss, limb weakness and dysarthria. Patients did not have visual complaints, but 12 out of 17 presented thickened retinal nerve fibers [72]. In France, a homozygous variant was found in two siblings with demyelinating motor-sensitive neuropathy (c.5744_5745delAT). Patients were also heterozygous for a larger *SACS* exon 10 deletion [73]. In a Finnish family, three heterozygous mutations were detected (p.E1100K, p.N1489S and p.M1359T). Patients presented spastic ataxia and distal weakness, and some family members were initially diagnosed with CMT. Sensorimotor demyelinating and cerebellar ataxia were common among them, and disease onset was between 6 and 25 years [74]. In Macedonia, one patient presented ataxia, gait, speech issues and mood swings at the age of 53, but depression appeared in her 30 s. She carried a homozygous *SACS* mutation, c.13721T>G;p. (F4574C), and developed cognitive decline [75].

**Table 2:** *SACS* mutation cases discovered in Europe. “hm” means mutation carried the homozygous form, “c het” means mutation had compound heterozygous allele. “rs” means respectively.

Mutation	Exon	Domain	AOO	Clinical Symptoms	Neurological Changes	Country (Ethnicity)	Refs.
c.1859insC, hm	8	SIRPT1	2 yrs	gait ataxia, dysarthria spasticity knee jerks mental retardation hypoacusis	nystagmus cerebellar atrophy in vermis severe loss of large myelinated fibers	Italy (Italian)	[48]
c.4999del CAGAA5000-(p.C1679X), hm	10	SIRPT2	before 10 yrs	ataxic gait tiptoeing dysarthric speech distal amyotrophy pes cavus leg stiffness urinary urgency dysphagia for fluid	severe spastic–ataxic gait cortical–subcortical atrophy severe atrophy of the upper cerebellar vermis		[47]
c.1858C>T (p.Q620X), c.4585insA:V1528→p.fsX1540), c.het	8 & 10, rs	SIRPT1 & SIRPT2, rs	before 2 yrs	delayed psychomotor development	atrophies in upper cerebellar vermis		
c.563G>A (p.G188E) + c.7394C>T (S2465L), c.het	7 & 10, rs	SIRPT1 & SIRPT3, rs	15 yrs	spastic–ataxic gait mild dysarthria leg weakness	mild cerebellar atrophy cerebellar vermis hypointense stripes in the pons		[49]
c.962G>A; p.R321Q + c.8330G>A, (R2777L), c.het	8 & 10, rs	SIRPT1 & SIRPT3, rs	15 mths	severe ataxia very slow ocular saccades severe dysmetria dysdiadochokinesia bilateral pes cavus	slight atrophy in superior cerebellar vermis corpus callosum thinning cervical spinal cord atrophy		
16 novel mutations, including a large deletion, hm or c-het	8 & 10	SIRPT 1-2-3 or XPCB or DNAJ	1–32 yrs	early-onset gait instability muscle tone can be decreased Babinski sign in all cases mild distal amyotrophy pes cavus urinary problems also appeared	superior cerebellar vermis hypointense stripes in the pons thinning corpus callosum		[50]
c.4198T>A/c.5719C>T, c.het	10	SIRPT2	15–16 yrs	progressive ataxia weakness spasticity gait limb deformities	atrophy in different brain areas: cerebellar vermis, hemispheres, corpus callosum thinning neuropathy		[51]
c.13132C >T (p.R4378X), hm	10	DNAJ	2 yrs	moderate ataxia mild dysarthria	NA		[52]
c.5629C>T- (p.R1877X), hm	10	SIRPT2	26 mths	severe ataxia moderate dysarthria	NA		[13]
c.600_604+1delAACAGG (p.I202fsX6), hm; 1.5 MB large deletion	8	SIRPT1	42 yrs	nystagmus, ataxic speech truncal & limb ataxia distal amyotrophy in all limbs limb deformities hearing loss	vermian atrophy increased optical disc area striking hypermyelinated retinopathy		[53]
c.6680T>C (p.L2374S), hm	10	SIRPT2	early childhood	developmental motor delay upper body ataxia, spastic paraparesis, limb deformities mild intellectual decline impaired night vision visual & hearing dysfunctions	cerebellar atrophy no hypermyelinated retinal fibers		[13]
c.10743C>T (p.Q3582X), hm	10	XPCPB	early childhood	delayed motor skills later a progressive spastic ataxia type 1 diabetes urinary retention abdominal pain	cortical & cerebellar atrophy thin corpus callosum		[54]
c.11471A>G, (p.N3799D), hm	10	SIRPT3	2.5–3.5 yrs	gait unsteadiness nystagmus, dysarthria distal amyotrophy spastic ataxia	retinal optic nerve hypermyelination cerebellar atrophy neuropathy	Turkey (Turkish)	[55,56]
c.9655_9658 delAGTT), truncation of I3199, hm	10	SIRPT3	1.5 yrs	gait unsteadiness nystagmus dysarthria distal amyotrophy spastic ataxia	axonal neuropathy retinal optic nerve hypermyelination		
c. 2018T>C (p.C648R), hm	8	SIRPT1	2.5 yrs	gait unsteadiness nystagmus dysarthria distal amyotrophy spastic ataxia	axonal neuropathy retinal optic nerve hypermyelination		
c. 8124delC-truncation of p.A2708, hm	10	SIRPT3	2 yrs	gait unsteadiness dysarthria distal amyotrophy spastic ataxia	axonal neuropathy retinal optic nerve hypermyelination		
c.1160A>G (p.F4011S), hm	10	between SIRPT3 & DNAJ	1 yr	ataxic gait spasticity dysarthria	retinal optic nerve hypermyelination cerebellar atrophy		[57]
c. 6945A>G (p.V3369A), hm	10	SIRPT3	3–7 yrs	dystonia, delay in motor development static cerebellar ataxia cerebral palsy	thick peripapillary retinal fibers cerebellar atrophy peripheral neuropathy		
c.12841T>A (p.K1404), c.6945A>G (p.V3369A), c.het	10	SIRPT2 & SIRPT3,rs	2 yrs	delay in motor development initially cerebral palsy	mild pes cavus		
c.6945A>G (p.V3369A), c.12020C (p.S4007F), c.het	10	SIRPT3 & loop between XPCB-DNAJ	4 yrs	delayed motor development spasticity initially cerebral palsy	peripheral neuropathy		
c.8346–8347insT (p.G2902V), hm	10	SIRPT2	2 yrs	delayed motor development cerebellar ataxia dysarthria ataxic gait initially hereditary spastic paraparesis limb deformity	mild pes cavus myelinated retinal fibers peripheral neuropathy		
c.5677G>A (p.R3801X), hm	10	SIRPT3	3 yrs	delayed motor development cerebellar ataxia dysarthria ataxic gait initially hereditary spastic paraparesis	peripheral neuropathy		
c. c.13485delC (p.K4495N), hm	10	DNAJ	3 yrs	delayed motor development initially cerebral palsy spasticity	peripheral neuropathy		
p.G2772A, hm	10	SIRPT3	early childhood	gait disturbance & paresthesia ataxia nystagmus limb deformities	atrophy in superior cerebellar vermis & in cervical spinal cord		[58]
c.2182C>T (p.R728), hm	8	SIRPT1	4 yrs	poor motor skills, tremor, ataxia, dysarthria, nystagmus spasticity in lower limbs global hyperreflexia pes cavus.	atrophy in cerebellar vermis		[59]
c.12461delC (p.P4154QfsX20), hm	10	between XPCB & DNAJ	After 1 yr	progressive spastic ataxia dysarthria tremor	atrophy of superior cerebellar vermis & cervical spinal cord mixed axonal demyelinating sensorimotor polyneuropathy		[60]
Several mutations, hm or c het	8 or 10	SIRPT2 or 3 or between SIRPT3 & DNAJ	1–13 yrs	patients had similar symptoms: cerebellar ataxia lower limb spasticity urinary dysfunction	sensorimotor axonal neuropathy cerebellar (vermis) atrophy	Netherlands (Dutch, British, Turkish)	[61]
c.3491T>A, p.M1164K, hm	10	SIRPT1	12–13 yrs	cerebellar ataxia with leg spasticity leg gait limb deformity	cerebellar atrophy particularly in the vermis no retinal hypermyelination	Belgium (Belgian)	[62]
Several mutations, hm or c -het	8 or 10, deletion of exon 3–5	SIRPT1 or 2 or 3 or HEPN	1–24 yrs	various symptoms ataxia gait dysarthria nystagmus dysmetria weakness	cerebellar atrophy vermis atrophy delayed myelination in pons or cerebellar areas some patients presented normal MRI	Belgium (Belgian, Moroccan, Serbian, Hungarian)	[63]
c.10517T>C (p.F3506S) + chromosomal deletion, c-het	10	SIRPT3	16 yrs	progressive spasticity ataxic gait muscle weakness mild dysarthria mild learning difficulties	atrophy of vermis superior superior cerebellar peduncles axonal sensorimotor polyneuropathy no retinal thickening		[76]
NA	NA	NA	1 yr	psychomotor delay non-progressive ataxia	myelinated retinal fibers atrophy in cerebellum	Spain (Spanish)	[77]
c.7848C>T (p.R2556C), hm	10	SIRPT3	before 1 yr	spastic truncal mild limb ataxia, slurred & scanning speech gaze-evoked nystagmus	cerebellar & retinal atrophy no hypermyelinated retinal fiber		[64]
c.826C>T (p.R276C) + c.3904C>T (p.P1302S), c-het	8 & 10, rs	SIRPT1+ between SIRPT3 & DNAJ, rs	early childhood	delay in motor skill development later gait instability slurred speech dysmetria cerebellar dysarthria	upper vermis & cervical spine atrophy bulky pons bilateral frontoparietal cortex atrophy sensory–motor polyneuropathy axonal demyelination		[65]
c.9695delC (p.T3232KfsX24),hm	10	SIRPT3	8–10 yrs	spastic ataxia gait disturbances muscle atrophy weakness	cerebellum atrophy hypo-intensities in the basis pontis	Greece (Greek)	[66]
c.9804_9805insC, (p.S3268 _Ifs), hm	10	SIRPT3	1.5–5 yrs	early onset ataxia dysarthria spasticity urinary dysfunction pes cavus	severe cerebella atrophy cervical spinal cord atrophy axon demyelination retinal optic nerve hypermyelination	Poland (Polish)	[67]
c.72276C>T (p.R2426X)	10	SIRPT3	32 yrs	typical ARSACS cognitive decline	NA	Russia (Russian)	[68]
c.13352T>C (p.L4451P) + c.6890T>G (p.L2297W), c.het	10	HEPN+ SIRPT2, rs	before 10 yrs	progressive lower limb stiffness gait unsteadiness dysarthria dysphagia urge urinary incontinence cognitive decline	cerebral & cerebellar atrophy	Norway (Norwegian)	[69]
17 novel *SACS* mutations, hm or c-het	8 + 9 + 10	SIRT1 or 2 or 3 or DNAJ or HEPN	1–30 yrs	delayed motor development gait ataxia other possible dysfunctions: nystagmus, dysmetria, incontinence, spasticity	neuropathy common cerebellar atrophy hypo-intensities in pons thinning corpus callosum possible cerebral cortex involvement	Germany (German, Turkish, Greek, Macedonian)	[4]
9 different mutations in 6 families, hm or c-het	8 or 10	SIRT1 or 2 or 3 or DNAJ or HEPN	2–15 yrs	non-syndromic hereditary motor & sensory neuropathy delayed early motor development tiptoe walking or gait instability slow disease progression	mixed demyelinating & axonal neuropathy MRI may be normal or cerebellar upper vermis atrophy	Germany (German, Italian, Romanian, Turkish, Arabic)	[70]
c.2076delG (p.T692) + c.3965_3966del (p.G1322Vfs*1343), c-het	8 & 10	SIRT1+ SIRPT2, rs	19–26 yrs	pyramidal signs spastic paraplegia saccade dysmetria amyotrophy distal weakness limb deformities pes cavus	atrophy of cerebellar vermis mixed demyelinating–axonal neuropathy no retinal hypermyelination	UK (British)	[71]
20 mutations, 11 novel mutations, hm or c-het	10	SIRT1 or 2 or 3 or DNAJ or HEPN	1–46 yrs	ataxia nystagmus, in all patients most patients have spasticity, sensory loss, limb weakness	thickening of the peripapillary retinal nerve fiber layer		[72]
c.5744_5745delAT (p.H1915Rfs*19),hm	10	SIRPT2	9–19 yrs	progressive walking difficulties fine motor skill disabilities balance dysfunctions intermittent falls the Achilles’ reflex decrease	peripheral neuropathy	France (French)	[73]
p.E1100K + p.N1489S + p.M1359T, c-het	10	SIRT1+ SIRPT2, rs	6–15 yrs	spastic ataxia weakness ataxic gait first diagnosis of Charcot–Marie–Tooth disease may be possible	sensorimotor demyelinating cerebellar atrophy cortical atrophy in frontal & parietal lobes	Finland (Finnish)	[74]
c.13721T/G (p.F4574C), hm	10	HEPN	37 yrs	progressive gait disturbance speech issues difficulties in walking slowness in daily activity mood swings, depression	bilateral cerebellar atrophy large arachnoidal cyst in posterior cranial fossa occipital bilateral cortical atrophy	Macedonia (Macedonian)	[75]

In Japan, *SACS* mutations were widely investigated (Table 3). Several Japanese cases presented similar phenotypes to the Quebec patients, as well as atypical cases, such as late-onset ataxia, ataxia without spasticity or retinal optic nerve hypermyelination. In 2004, a compound heterozygous sacsin mutation (C3774T, Q1198X) was discovered in a woman who developed spastic gait at the age of 9, which deteriorated in her 30s (clumsiness in hands, unsteadiness) [78]. Another compound heterozygous case of mutation was found in monozygotic twin sisters (c.2951_2952delAG+3922delT), who were unable to run fast and fell easily during their childhood. Their menstruation cycle became scanty and irregular, and pollakiuria appeared in them. No additional family member presented any neurological symptoms [79]. In 2005, two mutations, W395-fsX407 and V687-fsX713A, were found in a 25-year-old woman. The patient had slow gait progression, and she needed assistance with walking in her 20s. In addition, her speech became slurred. No myelinated retinal fiber was notable in her, and her motor nerve conduction became lower in several brain regions, including the median and ulnar nerves [80]. A homozygous deletion, c.6543delA, was discovered in two siblings with unique clinical symptoms, such as dementia, ophthalmoplegia, spasticity and amyotrophy. However, myelinated retinal fibers were absent [81]. Another homozygous two-base deletion, c.5988–9 del CT, was detected in a 29-year-old patient with spastic ataxic gait, distal muscle weakness and myelinated retinal nerve fibers in the retina [82]. Later, a sacsin T987C mutation was found in two patients without spasticity, but with a mild degree of ataxia in the form of slurred speech [83]. Additional atypical cases revealing an R2119* nonsense mutation were found in two Japanese siblings with spasticity and without retinal optic nerve hypermyelination [84].

In 2008, a compound heterozygous case of *SACS* N161fsX175 and L802P from two siblings with typical Japanese ARSACS patients was reported. Several family members with only one separate mutation were not affected [85]. In 2012, two novel mutations of compound heterozygous (c.[3769 G>T]+[11361–2insT] and c.[414 C>G]+[5263–4delAA]) were reported by Shizamaki et al. (2012) with age of onset at 12 years, and imaging analyses revealed atrophy in the cerebellum and pons [86]. In 2021, a compound heterozygous case with p.K4326E and p.L1412Kfs*16 mutations was discovered in a woman in her 40s, who had exhibited a slow progressive gait since childhood. Although she revealed the typical phenotype, it appeared to be milder than the typical ARSACS phenotypes. She did not present spasticity upon neurological examination, and the polyneuropathy was also missing [87,88].

Cases of ARSACS have also been discovered in other Asian countries, including China, Iran and Korea. The first Korean case of ARSACS was discovered in 2015 with a heterozygous deletion c.[4756_4760del] (p.N1586Yfx*3) in a 17-year-old female patient with classical early-onset spastic ataxia with sensorimotor polyneuropathy and distal amyotrophy. Since the patient was heterozygous with the novel deletion, it was possible that other mutations could be identified from the noncoding region (for example, splice site) or larger deletions [89]. In 2018, the second Korean case was found, in a patient who carried the compound heterozygous mutations c.8844delT (p.I2949Ffs*4) and c.11781_11782dupGC (p.P3928Rfs*17). The patient presented early-onset cerebellar ataxia, gait disturbances and weakness in the lower extremities. Neuroimaging and ophthalmologic analysis also supported the ARSACS diagnosis [90]. A recent case of another compound heterozygous *SACS* mutation, c.7272C>A:p.(C2424*) and c.11319_11321del:p.(R3774del), was found in a patient with cerebellar ataxia with migraine [91]. The patient developed gait disturbances in his teenage years, and his younger sister also developed similar symptoms [91].

In China, the first ARSACS case was discovered in 2016 in a patient with a compound heterozygous mutation, a c.11803C>T (p.Q3935X) variant, and a 1.33 megabase deletion of SACS [92]. The patient was diagnosed with an atypical case of ARSACS without spasticity in the legs or cerebellar ataxia. Instead, the clinical presentation involved early-onset peripheral neuropathy, mild spastic gait and horizontal gaze nystagmus [93]. Another case of spastic ataxia was discovered in 2018 in a patient with two novel variants, c.12637_12638delGA (p.E4213Rfs*3) and c.11274_11276delAAC (p.I3758_T3759delinsM) [93]. Each mutation was inherited from each unaffected parent. The patient presented cerebellar ataxia from his teenage years, followed by sensory–motor neuropathy, finger deformities and thickened retinal nerve fibers. Nerve conduction studies revealed that sensory action potentials were missing in all of his limbs, with reduced motor conductions [93]. Recently, a compound heterozygous mutation, c.8000T>C, p.F2667S and c.10685_10689del, p.F3562* mutations, were reported in a 30-year-old male patient with progressive ataxia without lower limb spasticity [94]. Additional Chinese cases of SACS mutations were also detected in ARSACS patients, such as p.P3007S + p.H3392fs; p.W1367X [95], p.T1746fs)+ p.I4362R [96], E1898X+ Y4225D or p.S578X + p.M2697Q fs*4 [97].

In Thailand, the first ARSACS patient was found at the age of 2, with progressive spastic ataxia from a homozygous mutation, c.382_383del (p.E128Sfs*2). [98] The patient presented several cerebellar dysfunctions and hypermyelination in the nerves of the optic disc. Cognitive functions remained normal [98].

In 2020, a homozygous frameshift mutation (c.5824_5827delTAC, a premature termination at residue 1942, p.Y1942Mfs*9) was found in an ARSACS patient from the Arabic peninsula (Kuwait) [99]. The patient presented a typical form of cerebellar ataxia, which also appeared in several family members (nephews, nieces). Balance disorder started from his teenage years. He also had a history of diabetes mellitus, retinal hemorrhage and transient ischemic attack prior to ARSACS [99].

An Iranian family presented atypical ARSACS with mirror movements, hypokinesia, bradykinesia and rigidity. Thickening in retinal axons was present in the affected patients. Affected family (female proband and her brother) members carried a novel homozygous mutation c.429_430delTT: p. W144VfsTer39 [100]. Another Iranian case revealed a homozygous A1373R mutation in a child, who was initially diagnosed with spinal muscular atrophy II (SMA-II). The significance of this mutation is currently unclear [101].

In recent years, a few ARSACS cases have been reported in Indian patients. The first case was found in 2014 in a patient with suspected Friedrich ataxia without spasticity and retinal fiber abnormalities initially, prior to a finding of a homozygous frameshift (c.14329fs*2725, p.R707Kfs*6) [102]. The second case was observed in 2017 with a 4-base-pair duplication in exon 10 (c.11690_11693dupGTGA; p.D3898EfsX2), in a patient with typical ARSACS symptoms: ataxia, motor dysfunctions, language impairment and hypermyelinated nerve fibers [103]. In 2019, another ARSACS case was found in a patient in his 20s from the Remote Tribal Jammu and Kashmir region, who had mild intellectual disabilities [104]. A homozygous frameshift mutation (C2869VfsX15) was detected in this patient. Imaging analyses revealed a hyperintense rim (“bithalamic stripes”) near the thalamic region [104]. In 2020, another homozygous deletion (c.8793 del A) was reported in a female patient from Kerala, who experienced difficulties in walking from her early childhood, followed by tremors and difficulties in holding objects [105]. One of her sisters also developed similar phenotypes [105]. Next, a compound heterozygous mutation, c.4232T C>G nonsense mutation and c.8132C>T missense variant, was reported in 2020 in a patient with progressive gait ataxia, dysarthria and lower limb stiffness [106].

Three ARSACS cases with homozygous *SACS* mutations c.2656C>T, c.4756_4760delAATCA and c.9119dupA were discovered in Pakistan [107]. The c.2656C>T and c.4756_4760delAATCA were associated with later-onset disease (11–12 years and 9–10 years, respectively), followed by cognitive decline with mental retardation [107]. Another patient with the c.9119dupA mutation presented typical ARSACS symptoms at the age of 1.5 years old [108].

In Israel, compound heterozygous mutations (sacsin D3269N and N2380K) were found in an ARSACS patient with ataxia and hearing impairment. A study with the patient’s fibroblasts revealed mitochondrial abnormalities, such as reduced numbers of mitochondria and an impaired mitochondrial network. An atypical symptom of retinal degeneration also appeared in this patient [109]. These mutations are shown in Table 3.

**Table 3:** *SACS* mutations discovered in Asia. “hm” means mutation carried the homozygous form, “c het” means mutation had compound heterozygous allele. “rs” means respectively.

Mutation	Exon	Domain	AOO	Clinical Symptoms	Neurological Changes	Country (Ethnicity)	References
p.3774C>T, p.Q1198X, c.het	10	XPCB & SIRPT1, rs	9 yrs	spastic gait in at 9 yrs old in her 30s, unsteadiness in gait hand clumsiness pes cavus optokinetic nystagmus	vermian atrophy in pyramidal system	Japan (Japanese)	[78]
c.2951_2952delAG(p.Q984GfsX986)+3922delT(p.1308LfsX1326), c. het	10	SIRPT1 + between SIRPT3 & XPBC, rs	15–20 yrs	gait & speech dysfunctions nystagmus pes caves cerebellar ataxia	atrophy in cerebellum cervical & thoracic cord progressive neuropathy, no hypermyelinated retinal fibers		[81]
c.32627-32636delACACTGTTAC(p.W395-fsX407), c.31760delT (p.V687-fsX713), c.het	8	SIRPT1	under 10 yrs	spasticity weakness in lower extremities limb & truncal ataxia pes caves pes vares	cerebellar atrophy (upper vermis) no retinal hypermyelination		[79]
c.6543delA, (p.R2002X), hm	10	SIRPT2	early childhood	spastic tetraplegia weakness & amyotrophy in limbs nystagmus ataxic speech pes cavus dementia	atrophy in superior vermis & thin corpus callosum no retinal myelinated fibers		[80]
c.5988-9del CT, hm	10	SIRPT2	early childhood	limb & truncal ataxia gait slurred speech limb deformities initial leg spasticity disappeared	cerebellar superior vermian atrophy decreased blood flow in cerebellum neuropathies hypermyelinated fibers in retinal optic nerve		[81]
c. 987C>T (p.F304S), hm	8	SIRPT1	before 10 yrs	gait in childhood worsened in their 20s no spasticity mild limb ataxia slurred speech	cerebellar atrophy myelinated retinal fibers		[82]
c. 6355C>T (p.R2119X), hm	10	SIRPT2	20s	nystagmus, ataxic speech truncal & limb ataxia limb deformities but no spasticity	cerebellar atrophy atrophy in mega cisterna magna & cervical cord, but not in cervical cord no retinal optic nerve hypermyelination		[83]
c.482delA (p.L802P), c.2405T>C (p.N161fsX175), c-het	10 & 7 rs	SIRPT1	late 10s- early 20s	mental retardation gait & speech disturbance nystagmus pes cavus no spasticity	progressive peripheral neuropathy cerebellar & cervical cord atrophy no myelinated retinal fibers		[85]
c.12976A/G (p.K4326Q), c.4233-4236 delACTT (p.L1412Kfs*16), c-het	10	DNAJ + SIRPT2, rs	~22 yrs	progressive gait disturbances saccadic eye movement nystagmus scanning speech cerebellar ataxia no spasticity	atrophy in cerebellar & cervical spinal cord area polyneuropathy no myelinated retinal fibers		[85]
c.3769 G>T (p.G1257X)+11361–2insT(p.R3788SfsX3820), c-het	10	SIRPT2 & SIRPT3, rs	12 yrs	cerebellar ataxia hyperreflexia, spasticity	cerebellar atrophy neuropathy myelinated retinal fibers		[86]
c.414 C>G (p.Y138X) +5263–4delAA (p.K1755VfsX1775), c-het	7 & 10, rs	SIRPT1 & SIRPT2, rs	12–19 yrs	cerebellar ataxia hyperreflexia spasticity unstable gait foot deformities stiffness spasticity in lower extremities ataxia in upper extremities muscle atrophy	cerebellar atrophy cervical cord atrophy spinal cord neuropathy loss of myelinated nerve fibers no myelinated retinal fibers		[87,88]
c.4756_4760del (p.N1586Yfs*3)+ putative noncoding mutation, c-het	10	SIRPT2	early childhood	slow progressive gait disturbance & dysarthria limb deformities pes cavus ataxia in limb spastic gait ataxia	hypointense stripes vertical hyperintensities in lateral pons atrophy in superior cerebellar vermis & cervical spinal cord myelinated retinal fibers	Korea (Korean)	[89]
c.8844delT (p.I2949Ffs*4) + c.11781_11782dupGC (p.P3928Rfs*17), c-het	10	SIRPT3 +between XPCB & DNAJ, rs	~20 yrs	early onset cerebellar ataxia gait disturbances weakness in lower extremities	cerebellar & spinal cord atrophy retinal nerve thickening		[90]
c.7272C>A (p.C2424X), c.11319_11321del (p.R3774del), c-het	10	SIRPT3 +XPCB, rs	~10 yrs	gait disturbances dysarthria & dysphagia	cerebellar atrophy retinal striations thickened retinal nerve fiber layer		[91]
c.11803C>T (p.Q3935X)+ 1.33Mb deletion, c-het	10	between SIRPT3 & XPCB	6 yrs	muscle atrophy weakness in distal extremities horizontal gaze nystagmus	cerebellar & spinal cord atrophy thickened retinal nerve fiber layer	China (Chinese)	[92]
c.12637 _12638delGA (p.Q4213Rfs*3)+ c.11274_11276delAAC (p.I3758_ TdelinsM), c-het	10	between XPCB & DNAJ + XPCB, rs	10’s	ataxia limb deformities	sensory–motor neuropathy thickened retinal nerve fibers		[93]
c. 8000T>C (p.F2667S), c. 10685_10689del (p.F3562X), c-het	10	SIRPT3 + between XPCB & DNAJ + XPCB, rs	early childhood	progressive cerebellar symptoms primarily affecting gait dysarthria dysmetria steppage gait pes cavus no spasticity	cerebellar atrophy & thinning of corpus callosum axonal neuropathy mild atrophy in cerebral cortex		[94]
c.5236dupA (p.T1746fs)+ c.13085T/G (p.I4362R), c-het	10	SIRPT2 +DNAJ, rs	NA	typical ARSACS ocular symptoms hearing loss	NA		[96]
c.9019C>T, p.P3007S and c.10174_10183del, p.H3392fs	10	SIRPT2 + between XPCB & DNAJ	early childhood	cerebellar ataxia pyramidal tract signs (lower limbs) dystocia at birth unstable gait incontinence epilepsy limb deformities	sensorimotor neuropathy dysplasia of corpus callosum upper cerebellar vermis atrophy thinning spinal cord swollen papilla		[95]
c.1773C>A (p.S578X) + c.8088_8089 insCA (p.M2697Q fs*43), c-het	8 & 10, rs	SIRPT1 + SIRPT3, rs	6 yrs	cerebellar ataxia reduced muscle strength	peripheral neuropathy cerebellar vermis atrophy hypo-intensities in pons		
c.5692 G>T, p.E1898X; c.12673-12677 del TATCA, p.Y4225D fs*6-c-het	10	SIRPT1 +DNAJ, rs	Early childhood	early onset cerebral ataxia slow speech, gait epilepsy limb deformities	Positive Babinski sign mild cerebral & severe cerebellar atrophy thinning spinal cord sensorimotor neuropathy		[97]
c.1773C>A, p.S578X; c.8088-8089 in. CA, p.M2697Q fs*4	10	SIRPT1 + SIRPT3, rs	6 yrs	unstable gait speech disturbances muscle weakness	moderate cerebellar atrophy neuropathy		
c.382_383del (p.Q128Sfs*2), hm	7	SIRPT1	2 yrs	nystagmus scanning speech finger dysmetria wide-based gait lower limb spasticity	hypermyelinated nerve fibers spinocerebellar cerebellar atrophy	Thailand (Thai)	[98]
c.5824_5827delTACT (p.Y1942Mfs*9), hm	10	SIRPT2	early teens	cerebellar ataxia limb deformities	demyelination & axonal loss cerebellar atrophy thinning corpus callosum	Kuwait (Kuwait)	[99]
c.429_430delTT (p.W144VfsX39), hm	7	SIRPT1	3 yrs	ataxic gait & dysarthria episodic muscle cramps mirror movements hypokinesia/bradykinesia rigidity	axonal–demyelinating sensorimotor neuropathy	Iran (Iranian)	[100]
c.4117_4118delGCinsAG (p.A1373R), hm	10	SIRPT2	early childhood	progressive muscle weakness poor growth initial diagnosis: SMA-II	NA		[101]
c.14329fs*2725 (p.R707Kfs*6), hm	10	SIRPT2SIRPT1	9–15 yrs	nystagmus pes cavus limb deformities postural tremor & instability no spasticity	sensory motor neuropathy no retinal fiber abnormality	India (Indian)	[102]
c.11690_11693dupGTGA (p.N3898QfsX2),hm	10	XPCB	4 yrs	delay in motor development speech was impaired dysmetria & ataxia in upper limbs loss of balance	cerebellar atrophy, “striped” pontine hypo-intensities myelinated nerve fibers in retina		[103]
c.8605delT (p.C2869VfsX15), hm	10	SIRPT3	14 mths	lowly progressive spastic–ataxic disorder mild intellectual disability	hypointense pontine stripes hyperintense lateral pons thickened retinal nerve fibers		[104]
c.8793 delA, hm	10	SIRPT1	early childhood	delay in motor development slurred speech saccades & broken pursuit movements gaze-evoked nystagmus	striped pons thinning corpus callosum bithalamic stripes		[105]
c. 4232T>G + c.8132C>T, c-het	10	SIRPT2 + SIRPT3, rs	3 yrs	progressive gait ataxia low limb stiffness dysmetria spasticity dysdiachokinesia nystagmus cerebellar gait	cerebellar atrophy striped pons peripheral neuropathy		[106]
c.2656C>T (p.Q886*), hm	10	SIRPT1	11–12 yrs	ataxias spasticity cognitive decline aggressivity seizures rapid progression intellectual disabilities	retinal thickening possible cerebellar atrophy hyperintensities in pons atrophic cerebellar hem spheres	Pakistan (Pakistani)	[107]
c.4756_4760delAATCA (p.N1586Yfs*3), hm	10	SIRPT2	9–10 yrs	ataxia spasticity slight cognitive decline intellectual disabilities	retinal thickening possible linear hypo-intensities in the pons		
c.9119dupA (p.N3040Kfs*4), hm	10	SIRPT3	1.5 yrs	spasticity spastic–ataxic gait bradykinesia mild dys tonic postures of upper limbs muscular atrophy	vermal & paravermal cerebellar atrophy thinning of corpus callosum global subcortical atrophy		[108]
p.N2380K & p.D3269N, c-het	10	SIRPT2 + SIRPT3, rs	16 mths	developmental delay nystagmus hearing impairment speech delay brisk deep tendon reflexes decrease in the number of cell mitochondria	retinal degeneration	Israel (Ashkenazi Jews)	[109]

The first case of ARSACS in the USA was reported in 2011, which was a compound heterozygous case (c. 3484 G>T, p. E1162X; and c.11707C>T, p. R3903X) detected in two Caucasian siblings (Table 4). Both siblings experienced clumsiness in walking, spasticity, and they were suggested to have spastic paraparesis initially. Due to the cerebellar atrophy, neuropathy in sensory motor neurons and possible autosomal recessive inheritance pattern, they were diagnosed with suspected ARSACS [110].

In 2013, a four-year-old child developed ataxia with delays in gross motor development and polyneuropathy. Whole-exome analyses revealed a homozygous mutation in SACS, 11637_11638delAG (p.R3879fs). In this family, consanguinity was present without a clear phenotype for ARSACS. Three of the patient’s cousins died with severe neonatal aspartylglucosaminuria [111]. Although a copy number variant (CNV) was found in one patient with multiple sclerosis (MS), features of ARSACS were prominent. This patient developed tremors and a mild balance disorder in their teenage years, with a history of learning difficulty and dyslexia. It could be possible that this individual carried both disorders [112].

Another case of ARSACS was detected in a male teenager with European heritage, who experienced stiffness in his legs and slow movement. At the age of 2, he was diagnosed with cerebral palsy and developed rigidity and bradykinesia later in life. He was also diagnosed with Parkinsonism. Although his speech production became slow, his language and intellectual abilities remained normal. The patient revealed a compound heterozygous known D1582N mutation and a frameshift c.7205_7206delTT (p.L2402Rfs*6). He also carried the A2510T variant with uncertain significance [113].

The first ARSACS case in the African American population was discovered in 2018, and the patient developed gait abnormalities and motor delay with low IQ. The patient also displayed headache and blurry vision. Array analysis revealed a 1.422 megabase loss in the chromosome 13q12.12 region, which contained the *SACS* gene. Another variant (c.11824dup) was detected in this patient. The maternal great grandfather of the patient also presented gait problems. Visual symptoms and seizures were prominent in several family members [114]. Fogel et al. (2012) analyzed several patients with sporadic ataxia, and discovered 11 *SACS* mutations in 39 patients, including p.N4573H, p.N4549D. A3927V or p.E174X. Majority of these patients presented spastic ataxia, but other disease phenotype also appeared, such as spinocerebellar ataxia, pure cerebellar ataxia or spastic paraplegia [115]

In Brazil, the first case appeared in a family with typical symptoms and neuroimaging features of ARSACS. Three siblings were affected, but the mutation was not found due to the absence of molecular genetic testing at that time [116]. In 2017, a case of homozygous c.5150_5151insA appeared in two female cousins of Germanic descent. Patients presented early-onset and slow progressive spastic ataxia. Retinal and nerve conduction abnormalities were also prominent [117]. In 2019, 13 Brazilian patients were investigated with ARSACS, who presented ataxia, spasticity and retinal nerve fiber thickening. Neuropathy and retinal abnormalities (peripapillary striations) or papillomacular fold were common in all 13 patients. Genetic analysis revealed 14 variants, among which two variants (p.L393Cfs*17 and N2760Mfs*6) were suggested to be novel [118].Two homozygous cases of p.R2703C and p.L308F were reported in 2017, in two unrelated patients. Both of these cases were associated with axonal CMT [119].

One mutation, c.7962T/G or p.(Y2654X), was reported in two Maori siblings with English ancestry from New Zealand (Table 4). They were initially suspected to have Friedrich ataxia, but no mutation was found in the FXN gene. Patients had lower limb weakness with upper limb ataxia. Symptoms appeared in their 20s and progressed further into their 40s [120].

**Table 4:** *SACS* mutations discovered in USA, South America and New Zealand. “hm” means mutation carried the homozygous form, “c het” means mutation had compound heterozygous allele. “rs” means respectively.

Mutation	Exon	Domain	AOO	Clinical Symptoms	Neurological Changes	Country (Ethnicity)	Refs
c.3484G>T (p.E1162X), & c. 11707C>T (p.R3903X), c-het	10	SIRPT1+ between SIRPT3 & XPBC, rs	2.5–3.5 yrs	poor motor skills gait ataxia spastic paraparesis tremor hands diminished muscle tone	cerebellar atrophy linear hypodensity in pons hypermyelination of retinal nerve fibers	USA (American)	[110]
c.11637_11638delAG (p.R3879fs), hm	10	SIRPT3 & XPBC, rs	4 yrs	delayed gross motor development ataxia gait lower galactocerebrosidase activity	sensorimotor demyelinating polyneuropathy linear hypo-intensities in pyramidal tract in the pons cerebellar atrophy		[111]
Chr13 duplication	NA	NA	13 yrs	mixed symptoms of ARSACS & MS, learning difficulties dyslexia	neuropathy, lesions in white matter mild cerebellar signs areflexia		[112]
c.4744G>A (p.D1582N) + c.7205_7206delTT (p.L2402Rfs*6), c-het	10	SIRPT2 + SIRPT3, rs	2 yrs	cerebral palsy slowly progressive muscle tone elevation rigidity bradykinesia speech became slower	retinal hypermyelination cerebellar atrophy thinning in corpus callosum	USA (American or mixed European)	[113]
c.11824dup (p.M3942Nfs*4), hm	10	XPCB	11 yrs	headache visual dysfunctions gait abnormality nystagmus saccadic dysmetria	asymmetric volume loss cerebellar vermis hypo-intensities in pons retinal hypermyelination	USA (African American)	[114]
11 *SACS* variants in 39 patients, hm or c-het	8 or 10	SIRPT 1-3, XPBC or HEPN	NA	28 patients with spastic ataxia one patient with spinocerebellar ataxia 2 with pure cerebellar ataxia 7 with spastic paraplegia	NA	USA (NA)	[115]
NA	NA	NA	early childhood	walking difficulties slurred speech ataxia, spasticity hyperreflexia dysarthria pes cavus limb deformity	cerebellar atrophy hypo-intensities in pons no retinal thickening	Brazil (Brazilian)	[116]
c.5150_5151insA	10	SIRPT2	early childhood	early-onset, slowly progressive spastic–ataxic disorder gait weakness in legs	mild parietal lobe atrophy cerebellar atrophy retinal nerve fiber hypermyelination	Brazil (German)	[117]
Several variants, including p.L393Cfs*17 & p.N2760Mfs*6	8 or 10, rs	SIRPT 1-3 or XPBC	1–44 yrs	ataxia, spasticity abnormal eye movement other symptoms: nystagmus, dysphagia, muscle cramps urinary dysfunction & epilepsy were also found	peripheral neuropathy was common thickened retinal nerve fibers cerebellar atrophy biparietal atrophy linear pontine hypo-intensities	Brazil (Brazilian)	[118]
c.8107C>T (p.R2703C) +c.922C>T-(p.L308F), hm	10 + 8, rs	SIRPT3 + SIRPT1, rs	8–9 yrs	axonal CMT disease limb deformities no cerebellar ataxia spastic paraplegia or intellectual disabilities	sensorimotor axonal neuropathy cerebellar atrophy demyelination no axonal thickening		[119]
c.7962T>G (p.Y2654X), hm	10	SIRPT3	20’s	cerebellar ataxia lower limb weakness dysarthria nystagmus	peripheral neuropathy minor cerebral atrophy some cerebellar atrophy	New Zealand (Maori & English)	[120]

## 5. Potential Involvement of *SACS* in Other Neurodegenerative Diseases

Currently, *SACS* has only been related to ARSACS. According to several studies on non-functional sacsin or sacsin knockout in cell lines or mice, sacsin is involved in various cellular roles, such as chaperon functions, mitochondrial mechanisms, microtubule filament control and cell adhesion [17]. It may not be ruled out that sacsin could exert an impact, directly or indirectly, in other types of neurodegenerative diseases (especially ataxias) than ARSACS. Since sacsin could interact with Hsp70 and ubiquitin proteasomes, its association may be involved in the defensive mechanisms against abnormal protein aggregations. When sacsin expression was attenuated, higher toxicity of repeat expansion was observed in comparison to normal sacsin expression. With Hsp70, sacsin may regulate the processing of ataxin-1 with polyglutamate expansion, especially the aberrant ataxin-1 degradation for protection against spinocerebellar ataxia-1 [2]. A putative association between sacsin and ataxin-3 was reported, where the N-terminal UbL domain of sacsin could directly interact with proteasomes. Since ataxin-3 could also interact with proteasomes, sacsin could affect the pathogenic mechanisms of ataxin-3 dysfunctions [121].

Interestingly, newly discovered *SACS* mutations in suspected patients with CMT-like neuropathy and atypical disease phenotypes suggest a potential pathological overlap between ARSACS and CMT [4].

The involvement of the *SACS* gene in other neurodegenerative diseases, such as AD, PD, ALS or CJD, has not been reported yet. Sacsin is closely involved in controlling the mitochondrial functions and dynamics. Sacsin dysfunctions have been associated with impairment of mitochondrial morphology, dynamics, organization and dysfunctions in Drp1, and could cause synaptic dysfunctions and loss of Purkinje cells [21,26]. In addition, the interactions between Drp1 and sacsin for proper mitochondrial functions, and Drp dysfunctions in the imbalance of mitochondrial fusion or fission, could also indicate the involvement of *SACS* mutations in AD, ALS, MS or PD. Altered Drp1 expression could cause the overexpression of amyloid beta, huntingtin or alpha synuclein. By interacting with Drp1, sacsin may influence the expression of different neurodegenerative-disease-related proteins indirectly. Defects in mitochondrial dynamics in other neurodegenerative disorders could be a common pathway associated with ARSACS and with *SACS* mutations. Hence, the contribution of sacsin in different neurodegenerative diseases could be hypothesized [122,123,124].

**Figure 5:**
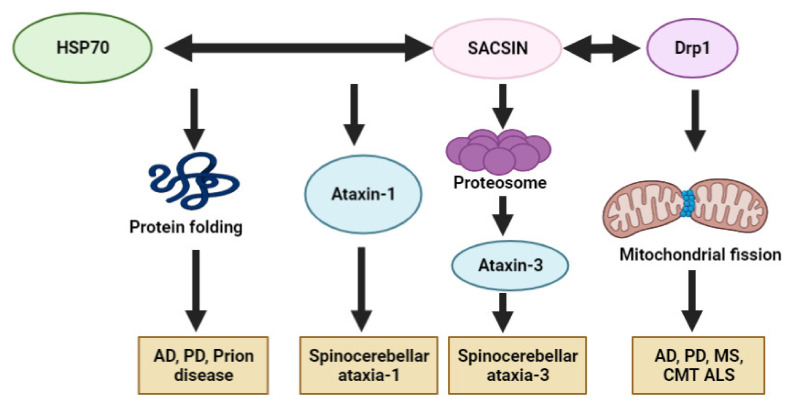
Possible disease mechanisms of sacsin protein in other neurodegenerative diseases: AD, PD, MS, CMT, ALS, SCA and CJD.

An additional putative common pathway between sacsin and other neurodegenerative diseases could be through its chaperon function. Sacsin contains homologous sequences with Hsp90. In addition, sacsin could interact with Hsp70 to exert several neuroprotective mechanisms [125]. Hsp proteins may play as a key role in protein folding and protect neurons against various protein-folding-related diseases. Impairment of Hsps and other chaperons could result in elevated oxidative stress and mitochondrial dysfunctions. Overexpressed Hsp70 was reported in AD mouse models, suggesting enhanced protective mechanisms by reducing APP cleavage and amyloid peptide production. Hsp70 could also enhance the transport of Tau protein and amyloid oligomers into proteasomes [126]. Hsp70 seemed to play an essential role in protecting against prion misfolding and aggregation [127]. In Hsp70 knockout mice, prion propagation and toxicity was accelerated [128]. Lastly, Hsp70 could block alpha synuclein oligomerization through a noncanonical site in the C-terminal domain to exert a protective function against PD and other synucleopathies [128]. These studies suggest that sacsin may contribute either directly or indirectly to neuroprotection against non-ataxia-related diseases. Figure 5 summarizes the possible common pathways between sacsin and other neurodegenerative diseases.

Additional evidence of an association involving common pathways between sacsin and other forms of neurodegenerative diseases was published by Morani et al. (2020) [36]. Their study analyzed proteomic data from ARSACS mouse models and isolated cells from ARSACS patients using SomaLogic technology. Several dysregulated pathways and differentially expressed proteins (DEPs) were found to be associated with neuroinflammation, synaptogenesis or cell engulfment. Several DEPs were found, which were involved in other neurodegenerative diseases: AD, PD, dementia with Lewy bodies and spastic paraplegia. Significant DEPs in ARSACS models were ephrins (*EFNB2*, *EPHA3* and *EPHB2*), *SNCA*, *APOE*, *ICAM5*, *SPHK1* and/or *STUB1*. This result points to the possibility of shared pathways between ARSACS and other neurodegenerative diseases. Hence, DEPs may act as risk factors or risk modifiers for ARSACS disease progression. Nevertheless, sacsin dysfunctions may alter the expression of ARSACS-related genes/proteins and may impact the pathological mechanisms of other neurodegenerative diseases, including AD, PD, ALS and CJD [36].

## 6. ARSACS Diagnosis and Potential Therapeutics

Genetic testing is required for the specific diagnosis of ARSACS. The standard Sanger sequencing would be challenging due to the size of the *SACS* gene. Hence, next-generation sequencing techniques would be the optimal approach in the discovery of novel causative genes or mutations in the *SACS* gene [129]. Additional biomarkers would be needed to enhance the disease’s diagnosis in combination with imaging biomarkers, such as brain and retinal imaging, in ARSACS diagnosis. Interestingly, “bithalamic stripes” detected by MRI imaging or retinal nerve fiber layer thickening could be an additional useful diagnostic biomarker of the disease [104].

Retinal nerve thickening was reported in several ARSACS patients. Recently, optical coherence tomography (OCT) was suggested to represent a significant diagnostic tool for investigating visual impairments and diseases in retina or neuropathy, as a non-invasive and cost-effective test. Hence, OCT could observe retinal nerve fiber thickening in patients with ARSACS [72,91]. Parkinson et al. (2018) performed OCT on patients with different types of ataxia (191) and controls (101). Retinal nerve fiber thickening was present only in ARSACS cases, and not in controls or other kinds of ataxia, such as Friedrich ataxia or spinocerebellar ataxia. This study proposed a cut-off value of 119 μm for the average retinal nerve fiber thickness. This value provided high specificity and sensitivity (100% and 99.4%, respectively) among ataxia patients [72]. Since the retina may be an excellent source of potential surrogate biomarkers in ARSACS, Rezende Filho et al. (2021) performed fundoscopy (another simple cost-effective technique) and OCT on patients with ARSACS and other forms of ataxia (spinocerebellar ataxia, autosomal recessive cerebellar ataxia, hereditary spastic paraplegia). The investigated retinal nerve fiber thickening in ARSACS by fundoscopy provided false negative data, suggesting that this method has lower sensitivity than OCT [130].

ARSACS patients presented other types of retinal abnormalities in comparison to control patients or those with other forms of ataxia. All ARSACS patients presented foveal hypoplasia, in addition to other impairments, such as retinal hyperplasia, sawtooth appearance or papillomacular fold. The above results suggest that monitoring neurophysiological abnormalities could yield promising biomarkers for ARSACS diagnosis [72,130], such as nerve conduction or nerve ultrasonography. Nerve enlargement and peripheral demyelination may be useful biomarkers in ARSACS [35,36]. Since the typical imaging biomarkers could be missing in some cases of ARSACS, Pilliod et al. analyzed 321 diagnosed patients with spinocerebellar degeneration from the SPATAX (http://spatax.wordpress.com accessed on 23 September 2021) database. They also collected fibroblast samples from skin biopsies in 11 ARSACS patients and 8 controls in order to perform mitochondrial morphology analyses, which indicated that mitochondrial abnormalities, such as bulbed mitochondria, were common among ARSACS patients. In addition, mitochondrial mass, oxygen consumption and the ratio of mitochondrial DNA/nuclear DNA were reduced among ARSACS patients. These anomalies in the mitochondrial network may be a useful diagnostic and prognostic biomarker to predict the pathogenicity of ARSACS [35]. Since spasticity is one of the typical key features, with reduced movement and coordination in patients with ARSACS, Lessard et al. performed the Lower Extremity Motor Coordination Test (LEMOCOT) for its possible usage in attempting diagnosis [131]. Analyzing pendulum oscillation amplitudes and their ratios using a wireless electro-goniometer provided information on the degree of spasticity in ARSACS patients. This device could effectively measure the evolution of spasticity in patients and provide an easy tool to compare the data from the pendulum between patients and unaffected individuals. Hence, this method would be an easy, rapid and cost-effective test for ARSACS diagnosis [132].

Since no therapy is currently available for ARSACS, the best disease management strategy is to mitigate the disease symptoms and improve the quality of the patient’s life. Positive associations were confirmed between fitness activities and the symptoms of neuromuscular/neurological diseases. An eight-week workout program, used by Audet et al. (2018), suggested that physical training may be beneficial for the fitness and functional capacity of ARSACS patients. Strengthened muscular and functional capacity was observed in patients who participated in the program. In addition, the regular workout improved patients’ ability to perform their daily activities. Furthermore, the reduced frequency of falls was notable. Regular training in patients with ARSACS could help them to retain and even regain their independence of movement [133]. Speech training was also effective to treat language impairments. Vogel et al. (2019) recruited ARSACS patients into a 4-week program with rater-blinded assessment of intelligibility. Although this was a preliminary study, the speech treatment improved the intelligibility of patients and enhanced the spontaneity of their speech [134].

Although no effective drug is available for ARSACS yet, certain candidate drugs may improve the quality of life of ARSACS patients. The main criterion for drug development is their capacity to cross the blood–brain barrier [135]. Docosahexaenoic acid (DHA), a dietary supplement, was investigated for its possible neuroprotective functions—namely, its anti-apoptosis, anti-inflammatory and anti-autophagic properties. Since DHA contains phospholipids, similar lipids to brain phospholipids, it was proposed in patients with ataxias with cerebellar and pyramidal involvement. Ricca et al. (2020) carried out a small clinical study in two ARSACS patients with SACS mutations. DHA was administrated orally for 20 months. Afterwards, investigators noticed stalled or slowed disease progression or the deterioration of clinical symptoms, with slight improvements in the functioning of the lower limbs and speech being reported, suggesting DHA as a safe and promising add-on therapy in patients with ARSACS and SACS mutations [136].

In addition, Idebenone (IDE), an analogue of coenzyme Q10, was successfully used to treat brain disorders with mitochondrial etiology by protecting against free radical toxicity. Since the solubility of IDE is low, many researchers have investigated it for the best drug delivery systems. Nanostructured lipid carriers (NLCs) loaded with IDE, with stability in water or cell media, and with successful penetration of the blood–brain barrier, were invented, representing a promising future approach in ARSACS therapy [135].

In sacsin knockout cells, the modulation of PTEN-FAK signaling could improve the ARSACS-related cellular dysfunctions, including microtubule organization, cell migration or adhesion [17]. PTEN has been verified as a negative regulator of focal adhesion. Hence, PTEN signal modulation has been suggested as a possible therapeutic target in ARSACS.

Since higher inductions of Hsp90 are observed in neurodegenerative diseases, a Hsp90 inhibitor was investigated for a possible reduction in disease progression and toxicity. A potential therapeutic candidate was KU-32, a Hsp90 inhibitor [137]. Hence, inhibiting Hsp90 was contemplated for its possible benefits in patients with ARSACS and different neurodegenerative disorders [138]. Ku-32 treatment improved the mitochondrial functions (electron transport, mitochondrial membrane potential) in cells among ARSACS patients [137].

## 7. Discussion and Future Insights

ARSACS appears to be one of the most common forms of ataxia besides Friedrich ataxia. Besides the French Canadian population, emerging cases of *SACS* mutations have been reported worldwide, including in Tunisia, Japan and Turkey. However, several cases of ARSACS may be unreported [34]. The typical disease phenotype of ARSACS includes the early onset of the disease, slow progression, cerebellar ataxia, spasticity, cerebellar atrophy, neuropathy, axonal demyelination and retinal nerve thickening. Additional symptoms, such as mental retardation, later disease onset and cognitive dysfunction, were also reported [19,68]. Interestingly, atypical cases of disease may also be possible (for example, lack of spasticity or retinal optic nerve hypermyelination), apart from the typical phenotype [4,83,85]. Additional atypical cases include patients with epilepsy, a CMT-like phenotype or hearing loss [4].

Homozygous or compound heterozygous mutations in *SACS* were associated with ARSACS. Noticeably, since clinical heterogeneities were observed, even from the same family, it may be possible that other genetic or environmental factors could impact the disease phenotype, besides the *SACS* mutations. Recently, the disease diagnosis became easier with the development of next-generation sequencing techniques, i.e., genome-wide and/or transcriptome-wide analyses, which could provide valuable insights into additional disease-modifying factors in ARSACS and or with *SACS* [38,129]. These genetic data could be correlated with imaging analyses, such as MRI [104] and OCT [72], to improve the disease’s diagnosis. Proteomic analysis would be a promising future investigation in understanding ARSACS and its progression and diagnosis [36].

Further studies are required to understand the functions of sacsin, especially in other neurodegenerative diseases. Although sacsin has been confirmed to be the causative factor of ARSACS, its impact on other diseases should be investigated, especially given the limited reports on its functions, which focus on chaperon interactions with Hsp70 and mitochondrial homeostasis [3,21]. Since the pathomechanisms of ARSACS may share similar pathways with other neurodegenerative diseases, such as AD, PD, ALS and CJD, it will be crucial to investigate the consequences of the gain or loss of functions [125,126,127,128,129]. The study by Morani et al. suggested that multi-omic (proteomic, genomic, transcriptomic) analysis in ARSACS models could be promising in the disease’s diagnosis, as well as in discovering the specific disease-causing pathways and risk-modifying factors, especially in the development of therapeutics [36].

## Data Availability

Not applicable.
